# Genotoxic Potential of Metodesnitazene and Etodesnitazene: Insights with and Without S9 Metabolic Activation

**DOI:** 10.3390/ijms27125360

**Published:** 2026-06-13

**Authors:** Francesca Rombolà, Dalila Maurizzi, Alessia Silla, Cristiana Caliceti, Sabrine Bilel, Patrizia Hrelia, Marco Malaguti, Monia Lenzi, Matteo Marti

**Affiliations:** 1 Department of Pharmacy and Biotechnology, Alma Mater Studiorum University of Bologna, 40126 Bologna, Italy; francesca.rombola3@unibo.it (F.R.); dalila.maurizzi@studio.unibo.it (D.M.); patrizia.hrelia@unibo.it (P.H.); 2 Department of Biomedical and Neuromotor Sciences, Alma Mater Studiorum University of Bologna, 40126 Bologna, Italy; alessia.silla2@unibo.it (A.S.); cristiana.caliceti@unibo.it (C.C.); 3 Department of Environmental and Prevention Sciences, University of Ferrara, 44121 Ferrara, Italy; sabrine.bilel@unife.it; 4 Department for Life Quality Studies, Alma Mater Studiorum University of Bologna, 47921 Rimini, Italy; marco.malaguti@unibo.it; 5 Department of Translational Medicine, Section of Legal Medicine, LTTA Center and University Center of Gender Medicine, University of Ferrara, 44121 Ferrara, Italy; matteo.marti@unife.it; 6 Collaborative Center for the Italian National Early Warning System (NEWS-D), Department of Anti-Drug Policies and Other Addictions, Presidency of the Council of Ministers, 00186 Rome, Italy

**Keywords:** metodesnitazene, etodesnitazene, new psychoactive substances, new synthetic opioids, genotoxicity, reactive oxygen species, in vitro mammalian cell micronucleus test, flow cytometry, chemiluminescence

## Abstract

The ongoing emergence of New Psychoactive Substances represents a growing threat to public health, as newly synthesized compounds continuously enter the illicit drug market, evading standard detection methods and challenging regulatory frameworks. Among New Psychoactive Substances, nitazenes are potent non-fentanyl opioids associated with severe cases of intoxication. This study evaluated the genotoxic potential of metodesnitazene and etodesnitazene in the human TK6 cell line. Cells were exposed to increasing concentrations of studied compounds, with and without S9 metabolic activation system. Preliminary assessments and micronuclei frequency analyses were performed by flow cytometry in at least three independent experiments. Metodesnitazene induced an increase in micronuclei frequency starting from 12.5 μM (*p* < 0.05), whereas etodesnitazene induced an effect only at 50 μM. Metabolic activation increases micronuclei formation at higher concentrations of metodesnitazene 25 μM, but did not substantially affect the response to etodesnitazene. Both compounds also induced intracellular reactive oxygen species production, measured through a chemiluminescent-based bioassay, suggesting oxidative stress as a potential contributing mechanism. These findings highlight the need for compound-specific toxicological profiling to better anticipate the acute and long-term risks associated with nitazene consumption.

## 1. Introduction

In a global context of increasingly stringent regulatory controls, the illicit drug market has adapted through the emergence of New Psychoactive Substances (NPS). These synthetic compounds are designed to mimic the effects of traditional drugs of abuse, with minor chemical modifications capable of preserving or even enhancing pharmacological potency [1], while remaining undetectable by standard toxicological assays and thereby complicating regulatory control [2].

Where possible, NPS are investigated to determine their pharmacological profile and abuse potential, providing essential data to support regulatory decision-making; accordingly, since 2008, the United Nations Office on Drugs and Crime (UNODC) has reported 1446 NPS identified across 153 countries [3,4].

Among the various classes of NPS, New Synthetic Opioids (NSO) have emerged as a particularly concerning group due to their high potency and risk of overdose [5]. They include fentanyl analogues, such as acetylfentanyl and 3-methylfentanylas well as non-fentanyl derivatives, namely MT-45 and brorphine [6,7].

NSOs exert their effects primarily through activation of the μ (MOR), δ (DOR) and κ (KOR) opioid receptors, leading to inhibition of central nervous system activity. The resulting clinical manifestations depend on receptor selectivity and on the amount of ligand reaching the target site and its affinity. Typical opioid-like effects include analgesia, sedation and euphoria, whereas adverse outcomes may involve respiratory depression and, in severe cases, life-threatening intoxication [8,9].

Within the broad group of non-fentanyl analogues, nitazenes represent one of the most alarming compounds. They were originally developed between the late 1950s and early 1960s by the Swiss pharmaceutical company Chemische Industrie Basel Aktiengesellschaft (CIBA). This research program, initiated even before the synthesis of fentanyl, sought to identify new analgesics that diverged structurally from traditional morphine-like opioids. Several molecules proceeded to early clinical testing, yet none achieved therapeutic approval due to severe adverse effects and a narrow therapeutic margin that offered no real advantage over morphine [10,11].

In recent years, illicit chemists have exploited early pharmacological research to revive nitazenes, producing new analogues at a rate that outpaces law enforcement efforts. As of February 2026, a total of 34 new variants have been notified to UNODC [12].

The main concern related to these compounds lies in their frequent presence in combination with other opioids within heroin mixtures; consequently, users are unlikely to be aware of consuming a nitazene, let alone the specific analogue involved [13]. Since 2023, the United Kingdom has reported outbreaks of nitazene-related overdoses, including more than 60 fatalities, linked to adulterated heroin and counterfeit oxycodone tablets [14]. Even more concerning is the detection of nitazenes in non-opioid mixtures, posing an additional risk of unintentional ingestion, particularly among opioid-naïve individuals [15].

Nitazenes are available in various forms, such as powders, counterfeit tablets, nasal drops or sprays and vaping liquids, and can be administered via multiple routes, such as injection, smoking, snorting or ingestion [16,17].

**Figure 1:**
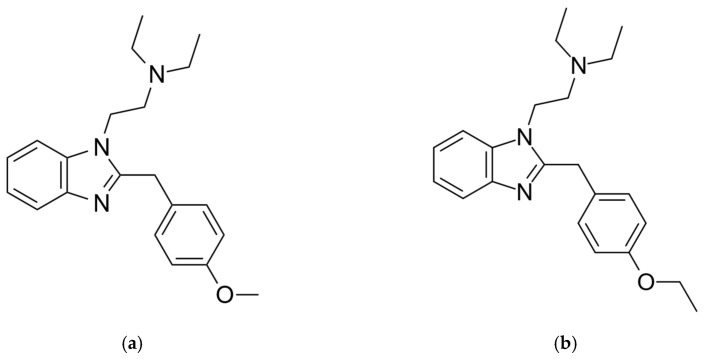
Chemical structures of (**a**) metodesnitazene and (**b**) etodesnitazene. The two compounds share the same core and differ solely in the substituent attached to the oxygen atom, a methyl and an ethyl group, respectively.

Regarding a structural perspective, nitazenes consist of a 2-benzylbenzimidazole scaffold, with a benzimidazole core substituted at the para position with a benzyl group. Structural variations are frequently observed at the 5-position of the benzimidazole ring, where a nitro group can be present, as in metonitazene and etonitazene, or absent, as in metodesnitazene (also known as metazene) and etodesnitazene (etazene), shown in Figure 1 [16,18].

The potency of these compounds varies significantly among analogues, as reflected in their pharmacodynamic profiles: nitazenes generally act as full agonists at MOR, binding at nanomolar concentrations, and as full or partial agonists at DOR and KOR. These properties reduce the clinical effectiveness of naloxone, the receptor antagonist used to treat opioid overdoses, often requiring higher or repeated doses for reversal [19].

Interestingly, an in vitro study from Vandeputte et al. (2024) [20] suggests that strong MOR binding does not always predict the degree of receptor activation. The overall pharmacological effects may also depend on factors such as bioavailability, blood–brain barrier penetration, metabolic stability and potential interactions with other receptors.

Based on the data provided by Kozell et al. (2024) [21], metodesnitazene and etodesnitazene show distinct interactions with the three opioid receptor subtypes. The latter binds to MOR with an affinity comparable to fentanyl, indicating strong receptor engagement, whereas the former shows lower MOR binding and requires higher concentrations to achieve similar effects. Both compounds have reduced affinity for DOR and KOR. In terms of potency, etodesnitazene is more effective than metodesnitazene at activating MOR and KOR. This finding is consistent with historical in vivo studies in mice conducted by CIBA, in which metodesnitazene displayed a relative potency comparable to morphine, while etodesnitazene exhibited 70-fold higher antinociceptive potency [11]. Nevertheless, several studies report that “desnitazenes” generally manifest a 10 to 100-fold reduction in potency compared with their 5-nitro counterparts [18,21,22].

In silico evaluations of absorption, distribution, metabolism and excretion indicate that etodesnitazene exhibits a bioavailability score of 0.55 and a lipophilicity approximately three times higher than that of morphine, which facilitates its absorption in the gastrointestinal tract and its penetration across the blood–brain barrier. It is not a substrate of P-glycoprotein and is predicted to inhibit multiple cytochrome P450 isoforms [23,24].

A recent study by Taoussi et al. (2024) [25] identified metabolites of metodesnitazene and etodesnitazene, totaling 10 and 22, respectively. The research was carried out by incubating the two substances with human hepatocytes and by analyzing postmortem biological fluids. The predominant metabolic reactions included N-deethylation and O-dealkylation, followed by O-glucuronidation. Minor pathways, such as hydroxylation and oxidative deamination, were also observed. Parent compounds were generally present at low levels in blood and urine, highlighting the importance of their metabolites as reliable biomarkers of exposure. In fact, due to their extreme potency, nitazenes are consumed in low doses, resulting in concentrations often below 10 ng/mL in biological matrices, posing challenges for toxicological detection, demanding highly sensitive analytical methods and likely contributing to the underestimation of nitazene-related intoxications and fatalities [25,26].

Because of their high abuse potential and the lack of accepted medical use, on 11 April 2024, etodesnitazene was added to Schedule I of the U.S Controlled Substances Act by the Drug Enforcement Administration (DEA), followed by metodesnitazene on 25 October 2024 [27,28].

Despite the registration of some nitazenes, experimental data regarding their toxicological profile, and particularly their genotoxic potential, remain extremely limited or completely lacking. This important gap in scientific knowledge contributes to a low awareness of the possible mutagenic and long-term health risks associated with these substances, not only among drug users but also within the general population [29].

In response to institutional early warnings, the characterization of emerging nitazene analogs has become an analytical priority. In a previous work, we characterized the behavior of metonitazene and etonitazene [29]. Building upon the previous findings, the present study focuses on evaluating how minor structural modifications in metodesnitazene and etodesnitazene influence their behavior. For the two molecules addressed in this manuscript, the only recent study available concerns etodesnitazene, which was found to induce a dose-dependent developmental toxicity in zebrafish, manifested as increased mortality, morphological abnormalities, delayed hatching and cardiotoxic effects [24].

To deepen the understanding of the health risks associated with these emerging synthetic opioids, this study investigated the genotoxicity of metodesnitazene and etodesnitazene in human lymphoblastoid TK6 cells following the Organisation for Economic Co-operation and Development (OECD) guideline No. 487 “In Vitro Mammalian Cell Micronucleus Test” [30]. As these substances undergo biotransformation in the human body, this research incorporated a metabolic activation system to evaluate possible genotoxic effects arising from their metabolites. Therefore, micronuclei (MNi) frequency was determined following a previously published flow cytometric protocol developed in our laboratory [31]. To better elucidate the molecular mechanisms underlying the observed genotoxic potential of these compounds, further analyses were carried out to assess the production of reactive oxygen species (ROS). While DNA is continuously subjected to a multitude of exogenous and endogenous genotoxic insults, intracellular ROS—frequently generated as by-products of mitochondrial respiration—represent a primary source of endogenous oxidative damage, leading to critical base lesions such as 8-oxoguanine (8-oxoG). Therefore, investigating ROS production was essential to determine whether oxidative stress serves as a key pathway mediating the genotoxicity of these compounds.

## 2. Results

In accordance with OECD guideline No. 487, cytotoxicity and cytostasis assays, supported by apoptosis evaluation, were performed to select the concentrations suitable for subsequent MNi frequency analysis [30]. These assessments were conducted first with the parent compounds and then repeated in the presence of metabolic activation. Lastly, the production of ROS was analyzed as a potential mechanism of DNA damage.

### 2.1. Cytotoxicity

Cells were treated with metodesnitazene and etodesnitazene concentrations of 12.5, 25, 50, 75 and 100 μM. After long treatment (26 h) exposure in absence of S9, cytotoxicity was evaluated staining necrotic cells with Propidium Iodide (PI), and data were normalized to the negative control (0 μM). As none of the tested concentrations induced cytotoxicity above the threshold of 55 ± 5% (corresponding to a cell viability of at least 45 ± 5%), all were considered for the following analyses.

**Figure 2:**
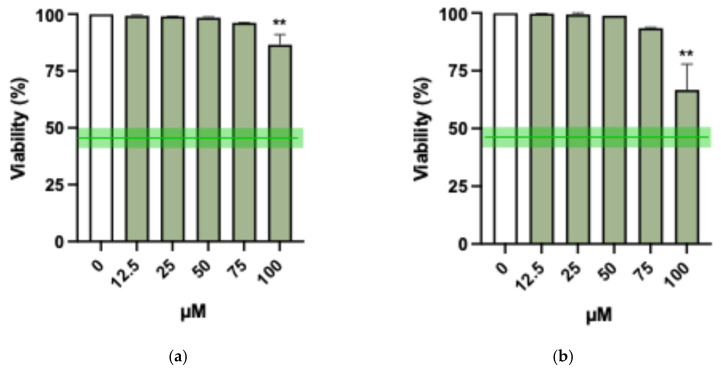
Viability of TK6 cells in absence of S9 after long treatment (26 h) exposure with (**a**) metodesnitazene and (**b**) etodesnitazene at the indicated concentrations compared to the negative control (0 μM). The green line represents the OECD threshold for cell viability. Each bar indicates the mean ± Standard Error of the Mean (SEM) of at least three independent experiments. Data were analyzed by Repeated Measures ANOVA followed by Dunnett post-test. ** *p* < 0.01 vs. 0 μM.

Figure 2 illustrates that, for both molecules, cell viability remained consistently above the OECD threshold indicated by the green line.

### 2.2. Cytostasis

Cytostasis was assessed by performing the same experiment as for cytotoxicity and was calculated using the relative population doubling (RPD), which provides an estimate of replicative activity in treated cultures compared to the negative control. According to the OECD guideline criteria, RPD values should not fall below 45 ± 5%.

**Figure 3:**
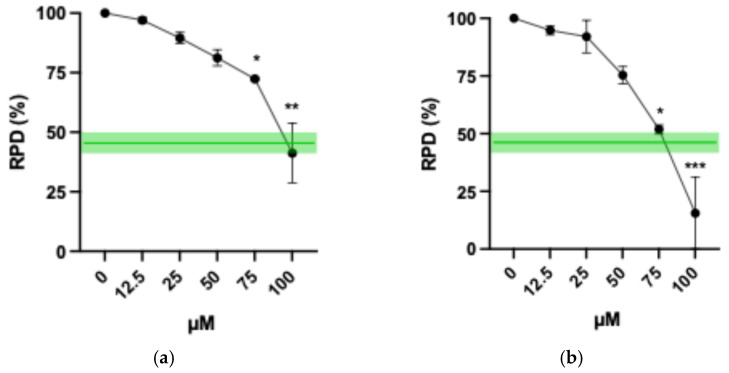
RPD of TK6 cells in absence of S9 after long treatment (26 h) exposure with (**a**) metodesnitazene and (**b**) etodesnitazene at the indicated concentrations expresses as percentage relative to the negative control (set to 100%). The green line represents the OECD threshold RPD. Each bar indicates the mean ± SEM of at least three independent experiments. Data were analyzed by Repeated Measures ANOVA followed by Dunnett post-test. * *p* < 0.05 vs. 0 μM; ** *p* < 0.01 vs. 0 μM; *** *p* < 0.001 vs. 0 μM.

As illustrated in Figure 3, at 100 μM the RPD dropped below the threshold for both compounds, leading to the exclusion of this concentration from MNi frequency analysis.

### 2.3. Apoptosis

PI staining alone allows discrimination between viable and necrotic cells, but early apoptotic cells may not be detected due to their intact membrane; for this reason, Annexin V Alexa Fluor 488 was also used to distinguish between early and late apoptotic cells.

Concentrations of 12.5, 25, 50 and 75 μM were tested, and those that induced more than a twofold increase in apoptosis compared to the negative control were excluded.

**Figure 4:**
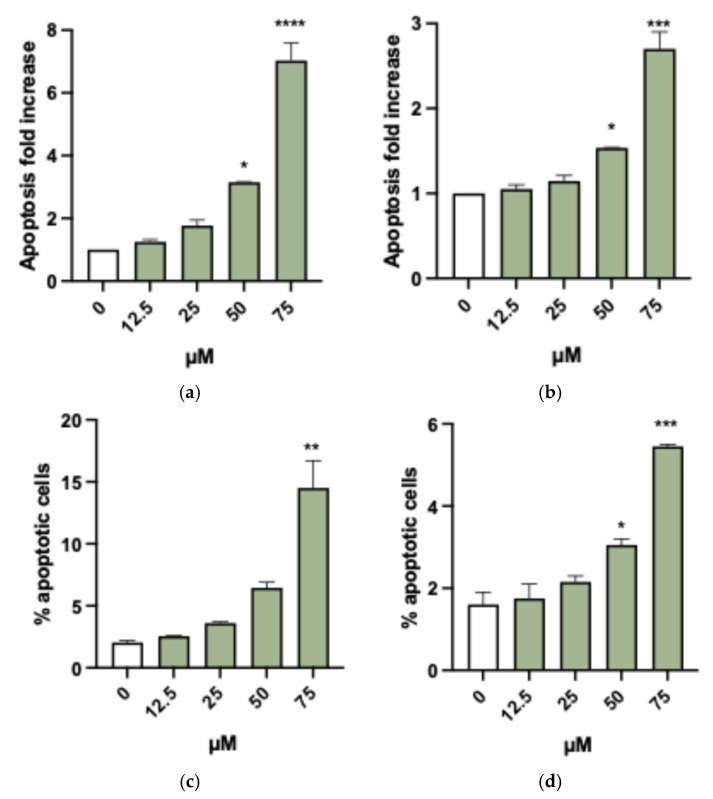
Apoptosis fold increase on TK6 cells in absence of S9 after long treatment (26 h) exposure with (**a**) metodesnitazene and (**b**) etodesnitazene at the indicated concentrations compared to the negative control (0 μM) set to 1. Graph (**c**,**d**) show a percentage of apoptotic cells following metodesnitazene and etodenitazene treatment. Each bar indicates the mean ± SEM of at least three independent experiments. Data were analyzed by Repeated Measures ANOVA followed by Dunnett post-test. * *p* < 0.05 vs. 0 μM; ** *p* < 0.01 vs. 0 μM; *** *p* < 0.001 vs. 0 μM; **** *p* < 0.0001 vs. 0 μM.

As shown in Figure 4, metodesnitazene exceeded this threshold at 50 and 75 μM, whereas etodesnitazene exceeded it only at 75 μM.

### 2.4. MNi Frequency

Based on the results of cytotoxicity, cytostasis and apoptosis assessments, the concentrations selected for MNi frequency evaluation were 12.5 and 25 μM for metodesnitazene and 12.5, 25 and 50 μM for etodesnitazene. The MNi quantification was conducted using SYTOX Green dye.

**Figure 5:**
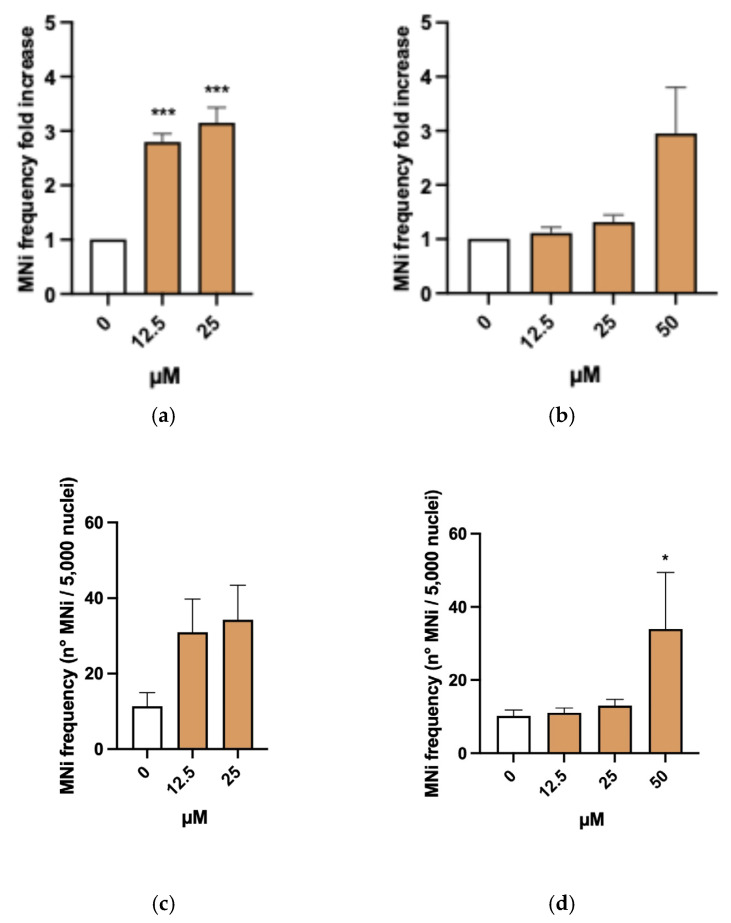
MNi frequency fold increase on TK6 cells in absence of S9 after long treatment (26 h) exposure with (**a**) metodesnitazene and (**b**) etodesnitazene at the indicated concentrations compared to the negative control (0 μM) set to 1. Graph (**c**,**d**) show an example of MNi frequency following metodesnitazene and etodenitazene treatment. Each bar indicates the mean ± SEM of at least three independent experiments. Data were analyzed by Repeated Measures ANOVA followed by Bonferroni post-test. * *p* < 0.05 vs. 0 μM; *** *p* < 0.001 vs. 0 μM.

In Figure 5, metodesnitazene increases MNi frequency already at 12.5 μM, inducing an increase higher than twice that of untreated cultures, with an even greater increase at 25 μM. In contrast, etodesnitazene treatment induces a double increase only at 50 μM.

### 2.5. Cytotoxicity, Cytostasis, Apoptosis and MNi Frequency with S9 Metabolic Activation

To obtain a more comprehensive assessment, the compounds were also evaluated in the presence of metabolic activation. For this purpose, the selected concentrations for cytotoxicity, cytostasis and apoptosis were 12.5 and 25 μM for metodesnitazene, 25 and 50 μM for etodesnitazene. Cells were exposed for 3 h in presence of S9 mix, followed by a 23 h recovery period in fresh medium.

**Figure 6:**
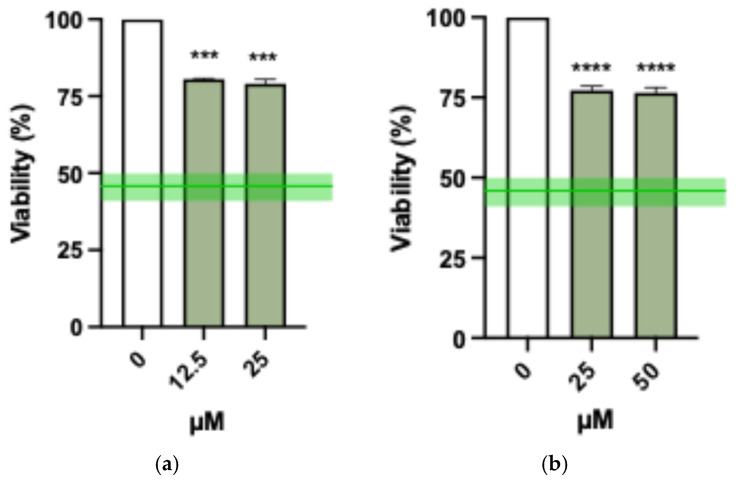
Viability of TK6 cells measured after short treatment (3 h) in presence of S9 with (**a**) metodesnitazene and (**b**) etodesnitazene at the indicated concentrations compared to the negative control (0 μM). The green line represents the OECD threshold for cell viability. Each bar indicates the mean ± SEM of at least three independent experiments. Data were analyzed by Repeated Measures ANOVA followed by Bonferroni post-test. *** *p* < 0.001 vs. 0 μM; **** *p* < 0.0001 vs. 0 μM.

As shown in Figure 6, the percentage of viable cells remained above the threshold for all tested concentrations. Compared to the data recorded in the absence of S9 mix, a slight decrease in viability was observed, which may be attributed to the metabolic activation system itself or to the formation of cytotoxic metabolites.

**Figure 7:**
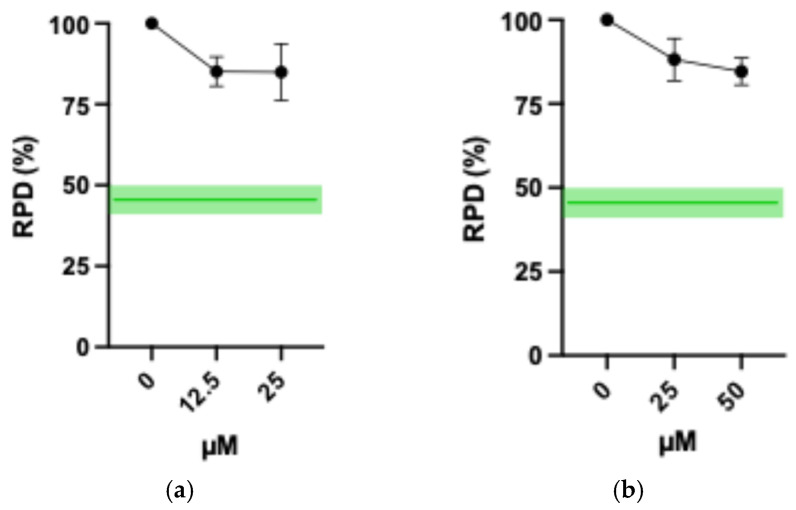
RPD of TK6 cells measured after short treatment (3 h) in presence of S9 with (**a**) metodesnitazene and (**b**) etodesnitazene at the indicated concentrations and expressed as percentage relative to the negative control (set to 100%). The green line represents the OECD threshold RPD. Each bar indicates the mean ± SEM of at least three independent experiments. Data were analyzed by Repeated Measures ANOVA followed by Bonferroni post-test.

Cell proliferation results are reported in Figure 7. Compared to the parental drug alone, cells treated with metodesnitazene in the presence of metabolic activation exhibited a slightly lower RPD. Etodesnitazene, on the other hand, produced similar values with or without S9 mix. In both cases, RPD remained above the OECD threshold, indicating adequate cell division.

**Figure 8:**
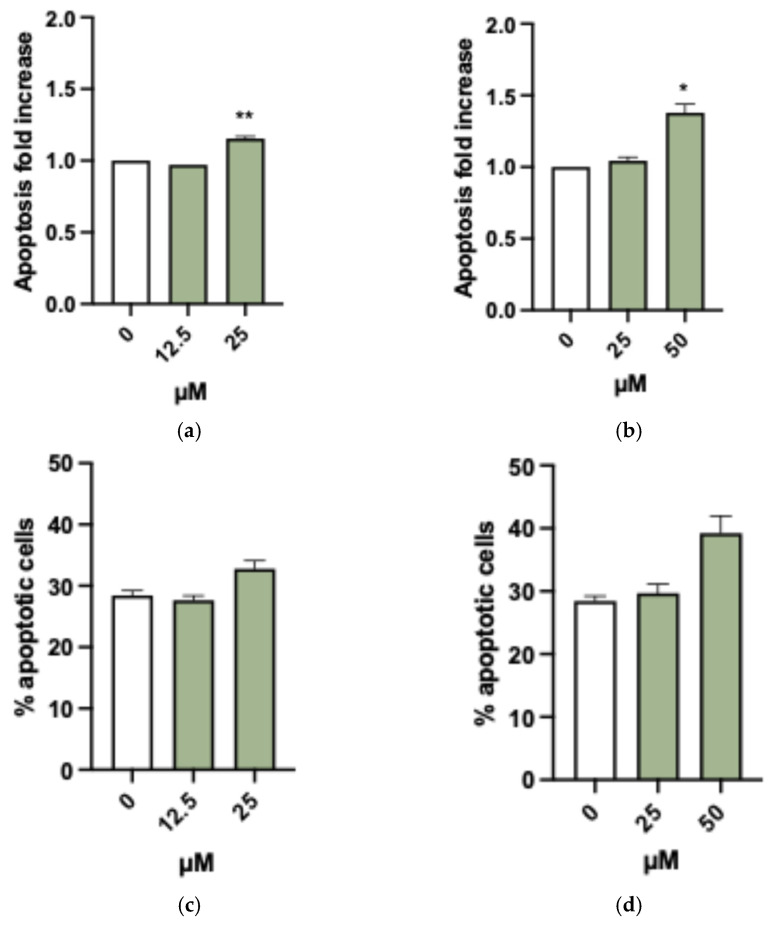
Apoptosis in TK6 cells in the presence of S9 metabolic activation. Graphs (**a**,**b**) show the fold increase in apoptosis following treatment with (**a**) metodesnitazene and (**b**) etodesnitazene at the indicated concentrations, compared with the negative control (0 μM). Graphs (**c**,**d**) show the percentage of apoptotic cells following the same treatments. Each bar indicates the mean ± SEM of at least three independent experiments. Data were analyzed by Repeated Measures ANOVA followed by Bonferroni post-test. * *p* < 0.05 vs. 0 μM; ** *p* < 0.01 vs. 0 μM.

Finally, Figure 8 indicates that apoptosis levels were comparable to those observed in the negative controls, confirming that in the presence of metabolic activation, the treatment did not induce excessive programmed cell death.

**Figure 9:**
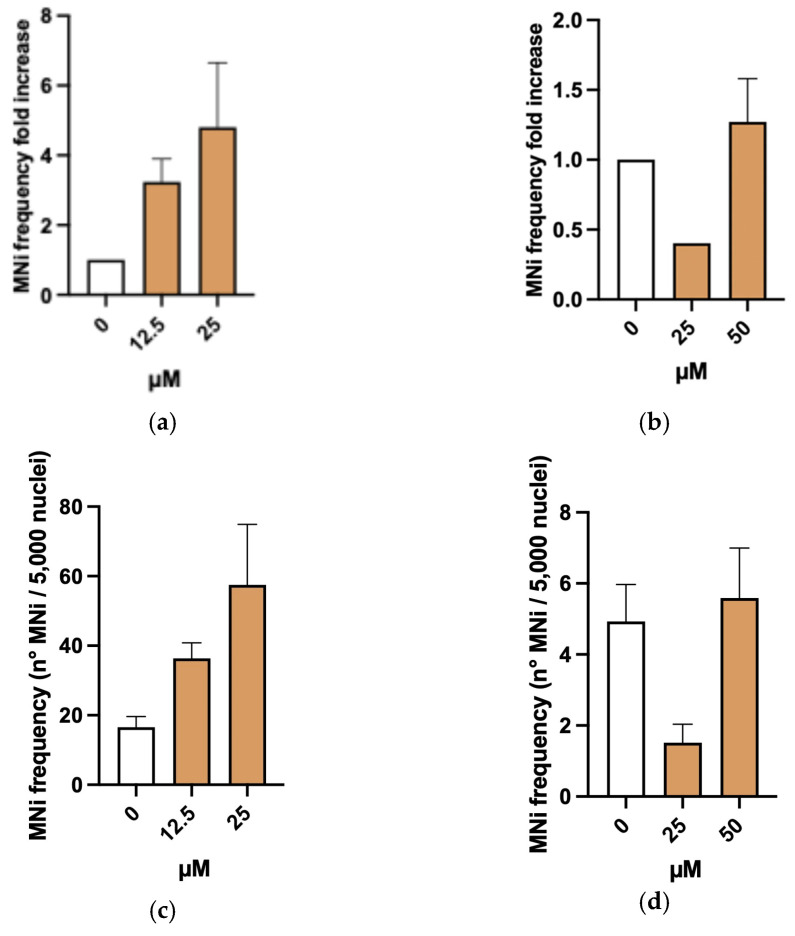
MNi frequency in TK6 cells in the presence of S9 metabolic activation. Graphs (**a**,**b**) show the fold increase in MNi frequency following treatment with (**a**) metodesnitazene and (**b**) etodesnitazene at the indicated concentrations, compared with the negative control (0 μM). Graphs (**c**,**d**) show the number of MNi out of 5000 nuclei following the same treatments. Each bar indicates the mean ± SEM of at least three independent experiments. Data were analyzed by Repeated Measures ANOVA followed by Bonferroni post-test.

Regarding genotoxicity, the results reported in Figure 9 revealed a partially different pattern from that observed in the parent compounds alone. Cultures treated with metodesnitazene in the presence of S9 mix showed no genotoxic effect at 12.5 μM, whilst a notable increase in MNi frequency was detected at 25 μM. In contrast, etodesnitazene under metabolic activation remained non-genotoxic at both tested concentrations.

### 2.6. ROS

As a final investigation, intracellular ROS production was assessed in cells exposed to the highest concentrations previously shown to induce genotoxicity, namely 25 μM metodesnitazene and 50 μM etodesnitazene. Hydrogen peroxide (H_2_O_2_) was used as a marker of intracellular ROS generation because of its relatively long half-life. The chemiluminescent (CL) signal elicited by the CL probe [32], which is proportional to H_2_O_2_ intracellular levels, was measured after 3 and 6 h of treatment.

**Figure 10:**
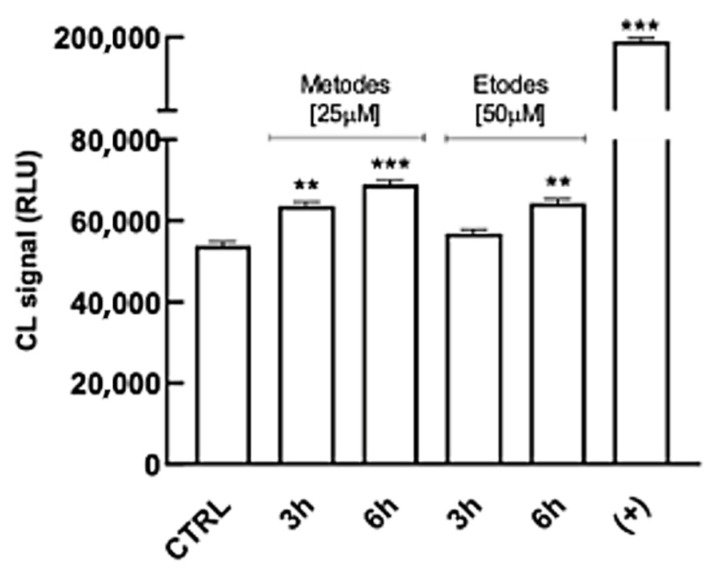
Chemiluminescence (CL) signal obtained for cells treated with metodesnitazene (25 μM) or etodesnitazene (50 μM) for 3 and 6 h and subsequently incubated with the CL probe (5 μM) for 20 min. Menadione (25 μM) was used as a positive control (+). The CL signal, proportional to intracellular H_2_O_2_ levels, was quantified as the area under the curve (AUC) over the 20–40 min interval. Each bar represents the mean ± Standard Deviation (SD) of three independent experiments. Statistical significance was determined by one-way ANOVA followed by Dunnett’s multiple comparison test versus untreated control (CTRL) ** *p* < 0.01 vs. 0 μM; *** *p* < 0.001 vs. 0 μM.

As illustrated in Figure 10, metodesnitazene induced a significant increase in the CL signal as early as 3 h, with a further enhancement observed at 6 h. In contrast, etodesnitazene did not produce a measurable increase at the 3 h time point, whereas a significant rise was detected following 6 h of exposure. As expected, a marked increase in the CL signal was observed in cells injured with menadione (25 μM), used as a positive control, thereby confirming the assay’s sensitivity to intracellular ROS levels.

Metodesnitazene and etodesnitazene both promote intracellular ROS production, with a faster response observed with the former and a delayed effect for the latter.

## 3. Discussion

The ongoing spread of nitazenes continues to raise major public health concerns. Given the structural diversity within this class and the possibility that even minor chemical modifications may markedly alter biological activity, compound-specific toxicological assessments are preferable rather than relying on analogies with other NSOs. This is especially critical as the toxicological profile of most NSOs remains narrowly focused on acute effects, leaving a significant gap concerning the long-term health consequences. Among these, genotoxicity warrants particular attention. In vitro genotoxicity assays such as the MN test provide evidence of a compound’s ability to induce chromosomal damage, a key event that may ultimately lead to stable genetic alterations. It is well established that mutagenicity represents a major mechanistic factor associated with an increased risk of several chronic diseases, including cancer and neurodegenerative disorders. Indeed, DNA damage and chromosomal alterations are widely recognized as important contributors to carcinogenesis and have also been implicated in the pathogenesis of a variety of neurodegenerative conditions [33].

In this regard, an extensive search of major scientific databases, such as PubMed, confirmed a substantial lack of studies addressing the genotoxic evaluation of NSOs, including nitazenes. Currently, our earlier findings represent the only available evidence in this field [29,34,35]. Since NSOs are frequently used chronically by dependent individuals, the absence of genotoxicity data means that a significant population may be unknowingly exposed to cumulative mutagenic risk. Thus, without comprehensive testing, these potential adverse outcomes remain unmonitored and overlooked in harm reduction strategies.

Aiming to overcome this knowledge gap, our previous research focused on the genotoxic assessment of several NSOs subclasses. We first evaluated fentanyl and its analogues (acrylfentanyl, furanfentanyl and ocfentanyl), finding that while fentanyl itself was non-genotoxic, its derivatives exhibited positive responses [34]. Our investigation then expanded to non-fentanyl analogues, specifically brorphine and its derivatives (orphine, chlorphine, iodorphine and fluorphine), where only brorphine and orphine were found to be non-genotoxic [35]. Most recently, we explored the nitazene class, testing metonitazene, etonitazene, isotonitazene and clonitazene; of these compounds, only the latter two demonstrated genotoxic activity [29]. Our findings confirm that even within the same class, NSOs can act through distinct mechanisms contributing to DNA damage and mutagenicity.

Building on these results, the present study was designed to evaluate the mutagenic potential of metodesnitazene and etodesnitazene, adhering to the standard hazard identification recommendations of OECD guideline No. 487 [30]. A flow cytometry-based protocol was chosen due to its clear advantages over traditional microscopy methods, which are limited by low cell counts, time-consuming analysis and operator-dependent variability. In contrast, the flow cytometric approach allows the rapid and accurate analysis of approximately 5000 cells within just two to three minutes, thereby improving efficiency and reproducibility [31].

Preliminary analyses indicate that both compounds remained within the acceptable cytotoxicity range across all concentrations. Respecting the threshold is crucial for maintaining biological relevance, as pronounced cell death or impaired proliferation can compromise the accurate and reliable determination of MNi. Cytostasis evaluation showed slowed cell cycle progression at the highest concentration (100 μM) for both substances, as the value was under the OECD threshold. Considering the results obtained on MNi frequency in subsequent assays, the lack of cytotoxicity and only mild cytostasis is particularly concerning, as it implies that cells are unable to counteract the activity of the two nitazenes. Cells may appear viable, proliferative and functionally unaffected, while nonetheless accumulating genetic damage that is not effectively repaired and can be transmitted to daughter cells.

The guideline also recommends the assessment of additional markers of cellular stress to complement the primary cytotoxicity evaluations, provided they are not used as a substitute for the standard measurements [30]. In this study, apoptosis was selected as an additional endpoint to prevent apoptotic bodies from being erroneously scored as MNi, which could lead to false-positive results. Accordingly, a two-fold increase relative to the control was considered beyond the acceptable threshold. Based on this criterion, metodesnitazene at 50 and 75 μM and etodesnitazene at 75 μM surpassed this limit. Although these concentrations did not induce detectable cytotoxicity, the activation of programmed cell death suggests that the defensive mechanism can identify and eliminate cells deemed unsuitable for survival. However, while the induction of apoptosis at high doses serves as a positive indicator of cellular competence, it is not efficient enough to prevent genotoxic effects at lower concentrations.

These evaluations allowed the selection of concentrations suitable for MNi analysis. A consistent dose-dependent trend was observed across independent experiments compared to the concurrent negative control. In particular an increase in MNi frequency was detected in treated cells at both concentrations of metodesnitazene (12.5 and 25 μM) and at the highest concentration of etodesnitazene (50 μM), with values approximately two-fold higher than those of the untreated control. Despite such increase, consistently exceeding a twofold change, statistical significance was not always reached (most likely because of the high SEM values, associated with these datasets.). This aspect remains relevant and represent the main limitation of this study and requires further investigation.

However, this trend in MNi frequency increase gain further importance when considered in relation to our previous results on metonitazene and etonitazene, for which no genotoxic effects were detected under comparable experimental conditions [29]. The contrasting behavior observed between these compounds and their corresponding desnitro suggests a possible structure–activity relationship within this class. In particular, the absence of the 5-nitro group appears to be associated with the emergence of a mutagenic response, hinting that even subtle structural modifications could critically influence the genotoxic profile of nitazenes, potentially altering their interactions with cellular targets. However, this hypothesis requires further studies. Considering these results, the effects linked to metabolites formed under metabolic activation were also investigated to better mimic in vivo conditions. Since the cytotoxicity, cytostasis, and apoptosis threshold values were respected, MNi frequency was assessed at the same concentrations used in the experiments without metabolic activation. In presence of S9 mix, only cells treated with metodesnitazene at 25 μM showed a two-fold increase in MNi frequency compared with the negative control.

The study by Taoussi et al. (2024) [25], conducted in vitro on human hepatocytes and post-mortem biological fluids (supported by in silico predictions), identified 10 metabolites of metodesnitazene and 22 metabolites of etodesnitazene. While the use of an exogenous metabolic activation system provided a broader toxicological evaluation, the specific metabolites formed and their individual effects are unknown. This represents a limitation of the current study, as the observed genotoxic responses were elicited by a complex pool of metabolic products rather than a single identified molecule. Further investigations using isolated metabolites would be necessary to pinpoint the exacts molecular entity responsible for DNA damage.

In presence of S9 mix, cell viability decreased to approximately 75% already at the lowest treatment concentration compared with the S9-matched control, suggesting a possible effects of metabolite generation. Cytostasis results also showed a general reduction in RPD, suggesting that metabolites may induce a mild cell-cycle slowing, which at higher concentrations could progress to arrest. Interestingly, apoptosis induction decreased under metabolic activation for all tested concentrations and compounds. Regarding genotoxic analysis, metodesnitazene alone showed an increase in MNi frequency at both concentrations tested. In presence of S9 metabolic activation, the 12.5 μM concentration yielded a result comparable to the negative control, whereas the 25 μM concentration showed an increase approaching four-fold relative to control levels. Out of the three concentrations tested, etodesnitazene alone showed an increase in MNi frequency only at the highest concentration; whereas under metabolic activation, the 25 μM concentration resulted in a reduction compared to the negative control, while the 50 μM showed a slight increase, which remained within the range of variability of the control values. At least three independent experimental replicates were performed, fully adhering to the standard hazard identification recommendations of OECD Guideline No. 487. While statistical significance was not systematically achieved across all tested concentrations and the graphs show wide error margins, the overall evaluation of the genotoxic potential was based on biological relevance and trend consistency across the independent runs, as suggested by international regulatory frameworks. The dose-dependent upward trend observed across all independent replicates warrants consideration as a biologically relevant signal, albeit descriptive and requiring further mechanistic validation. Furthermore, this apparent variability is indicative of the stochastic nature of genotoxic events. Therefore, the present findings indicate that both metodesnitazene and etodesnitazene may be associated with genotoxic responses in TK6 cells. The data also suggest a possible modulation of the observed effects by metabolic activation; however, these results should be interpreted with caution given that not all effects reached statistical significance and the mechanistic interpretation remains preliminary. Indeed, the genotoxic potential of a substance can manifest through various molecular pathways, ranging from the formation of covalent DNA adducts to the interference with the mitotic spindle apparatus and the induction of oxidative stress [33]. In this study, we aimed to investigate the latter mechanism; specifically, TK6 cells were treated with 25 μM metodesnitazene or 50 μM etodesnitazene, and intracellular ROS production was evaluated using a CL-based bioassay. Metodesnitazene induced a significant increase in ROS at both time points. Etodesnitazene, though, generated a notable rise only at 6 h. Collectively, these results indicate that both compounds promote intracellular ROS generation, with metodesnitazene eliciting a more rapid pro-oxidant response and etodesnitazene displaying a delayed effect. While the observed increase in ROS levels supports oxidative stress as a possible contributor to the trend in MNi frequency increase induced by metodesnitazene and etodesnitazene, our data do not establish a direct causal or quantitative relationship between ROS production and MNi frequency. Menadione was used only as a positive control for the ROS assay and not as a reference compound for genotoxic potency [36,37,38,39]. Accordingly, these findings should be interpreted as suggesting a possible involvement of oxidative stress rather than proving a causal mechanism. Future studies employing antioxidant co-treatment, oxidative DNA damage markers, and/or metabolite characterization will be required to further clarify the mechanistic relationship between oxidative stress, biotransformation, and genotoxicity [40]. These investigations fall outside the scope of the present study and represent valuable directions for future research.

Although illicit substances are subject to strict regulatory controls and are not intended for use, their consumption nonetheless persists worldwide, emphasizing the urgent need for a solid scientific understanding of the associated health impacts. This study demonstrates that both metodesnitazene and etodesnitazene may induce genotoxic damage in TK6 cells, with the magnitude of the response influenced by metabolic activation. From a public health perspective, these findings strengthen the growing concern surrounding nitazenes, which are increasingly found on the illicit drug market and frequently consumed unknowingly or in combination with other psychoactive substances [13]. In this context, the identification of genotoxic effects raises important questions regarding potential long-term health consequences in exposed individuals. Unlike acute toxicity, which is often associated with respiratory depression and overdose, genotoxicity may remain clinically silent while contributing to cumulative DNA damage. In a broader biological framework, persistent macromolecular damage is theoretically associated with an increased long-term risk of chronic-degenerative pathologies [19,41]. However, it must be emphasized that the concentrations utilized in this screening were selected in strict accordance with the OECD Guideline No. 487 requirements, which mandate testing up to cytotoxic levels to ensure reliable hazard identification. Since these experimental conditions reflect a standard high-dose regulatory screening rather than actual physiological exposure levels, these potential long-term outcomes must be interpreted with caution.

Moreover, the variability observed among structurally related compounds complicates risk assessment, limiting the ability to predict the safety profile of newly emerging derivatives. A key objective for future research will be to identify the specific metabolites responsible for the genotoxic effects observed under metabolic activation. Finally, further efforts in toxicological profiling, including chronic exposure investigations, will be essential to limit the harm associated with these substances as their presence in the illicit drug market continues to expand.

## 4. Materials and Methods

### 4.1. Reagents

Ethylenediaminetetraacetic acid (EDTA), ethanol, L-glutamine, penicillin-streptomycin solution (PS), potassium chloride, potassium dihydrogen phosphate, Roswell Park Memorial Institute (RPMI) 1640 medium, fetal bovine serum (FBS), sodium chloride, sodium hydrogen phosphate and menadione were all purchased from Merck (Darmstadt, Germany). Annexin V Alexa Fluor 488 Ready Flow Conjugate, Annexin Binding Buffer (5X), propidium iodide (PI), RNase A, SYTOX Green were purchased from Thermo Fisher Scientific (Waltham, MA, USA). Mutazyme 10% S9 mix was purchased from Trinova Biochem GmbH (Giessen, Germany). The CL probe (AquaSpark™510 Peroxide Probe) was provided by BiosynthCarbosynth (Staad, Switzerland).

### 4.2. Nitazenes

Metodesnitazene and etodesnitazene were obtained from LGC Standards S.r.L (Milan, Italy). Stock solutions 10 mM were prepared by dissolving the compounds in absolute ethanol and stored at −20 °C until use. Both solutions were handled under light-protected conditions. To minimize potential solvent-related toxicity, the final concentration of absolute ethanol was maintained below 1% *v*/*v* in all experimental conditions.

### 4.3. Cell Culture

This study was conducted using the TK6 cell line, a p53 competent human lymphoblastoid model characterized by a low rate of spontaneous mutations and a favorable replication cycle of 13 h [42]. It is widely recognized as a reliable system, being included among the cell lines validated by the OECD for assessing MN frequency [30]. 

TK6 cells were purchased from Sigma-Aldrich (St. Louis, MO, USA) and grown in complete medium consisting of RPMI-1640 supplemented with 10% FBS, 1% L-glutamine and 1% PS. Cell cultures were maintained at 37 °C in a humidified atmosphere containing 5% CO_2_. To ensure optimal exponential growth, cells were periodically diluted with fresh medium, never exceeding the critical density of 1 × 10^6^ cells/mL.

### 4.4. Test Conditions

In accordance with OECD guideline No. 487, TK6 cells were exposed to the two nitazenes in absence of S9 for long treatment, i.e., 26 h, corresponding to approximately 1.5–2.0 cell division cycles [30].

Given the limited metabolic competence of this cell line, experiments were also conducted for short treatment, i.e., 3 h, in the presence of a human-derived S9 metabolic activation system to enable a more comprehensive evaluation. The S9 mix consists of a post-mitochondrial liver supernatant containing both microsomal and cytosolic enzymes involved in phase I and phase II metabolism. It was added to the cell cultures at a final concentration of 1% for 3 h, after which cells were incubated with fresh medium for an additional 23 h, as recommended by the guideline [30].

#### 4.4.1. Selection of Concentrations

The study was initiated using a concentration range of 0–100 μM, with the highest concentration corresponding to the maximum permitted level of organic solvent (1% *v*/*v*). Lower concentrations were defined by successive twofold dilutions, with an intermediate point of 75 μM included between 50 and 100 μM due to the substantial difference. 

Cytotoxicity and cytostasis were evaluated at all concentrations and compared with the OECD guideline No. 487 thresholds of 55 ± 5%. The guideline also allows the assessment of additional cytotoxicity markers; apoptosis was selected for this purpose [30].

#### 4.4.2. Measurement of Cytotoxicity

For the evaluation of cytotoxicity in the absence of metabolic activation, aliquots of 2.5 × 10^5^ TK6 cells were exposed to the compounds at increasing concentrations ranging from 0 to 100 μM for 26 h. In the presence of S9 mix, cells were treated with metodesnitazene at 0, 12.5, and 25 μM, or with etodesnitazene at 0, 25, and 50 μM. In this case, after 3 h of metabolic activation, the S9 mix was removed by washing and the cells were resuspended in fresh complete medium for a further 23 h.

At the end of the treatment period, cytotoxicity was assessed by cell viability using PI, which discriminates between viable and necrotic cells based on the intensity of red fluorescence. For each sample, 1000 events were acquired using GuavaSoft™ 4.5 software (Cytek Biosciences Inc., Fremont, CA, USA), and cell viability was automatically calculated based on these events.

#### 4.4.3. Measurement of Cytostasis

Cytostasis was evaluated under the same experimental conditions described for cytotoxicity. Using GuavaSoft™ 4.5 software (Cytek Biosciences Inc., Fremont, CA, USA), population doubling (PD) was calculated based on the ratio between the number of cells detected by flow cytometry and the number of seeded cells, as a measure of cell proliferation (Equation (1)).

(1) $$PD=\frac{1}{\log2}\times \log\frac{Final\;number\;of\;cells}{Starting\;number\;of\;cells}$$

Subsequently, PD values obtained for each treated condition were compared with that of the negative control to calculate the RPD, in order to verify that the majority of cells had completed at least 1.5 cell division cycles (Equation (2)).

(2) $$RPD=\frac{PD\;in\;treated\;cultures}{PD\;in\;control\;cultures}\times 100$$

#### 4.4.4. Measurement of Apoptosis

Apoptosis was investigated as an alternative mechanism of cell death to necrosis by measuring the increase in apoptotic cells in treated samples compared with the negative control. Concentrations within the 0–100 μM range that met the threshold criteria from previous cytotoxicity and cytostasis assays were selected for testing. In the absence of metabolic activation, concentrations ranging from 0 to 75 μM were evaluated for both compounds, whereas under metabolic activation metodesnitazene was tested at 0, 12.5 and 25 μM and etodesnitazene at 0, 25 and 50 μM.

Aliquots of 2.5 × 10^5^ TK6 cells were treated for 26 h without S9 mix, or for 3 h with S9 mix followed by a 23 h recovery period. At the end of the treatment, cells were stained with Annexin V Alexa Fluor 488 in combination with PI, allowing discrimination between viable, apoptotic and necrotic cells. This approach also enabled distinction of apoptotic stages: cells in the early stage displayed only green fluorescence from Annexin V Alexa Fluor 488 binding to externalized phosphatidylserine, whereas cells in the late stage exhibited both green and red fluorescence, due to the loss of membrane integrity, which allowed PI to enter the cell and intercalate with chromatin.

A total of 2000 events per sample were acquired, and GuavaSoft™ 4.5 software (Cytek Biosciences Inc., Fremont, CA, USA) automatically calculated the percentages of viable, apoptotic and necrotic cells.

#### 4.4.5. Measurement of MNi Frequency

For MNi frequency analysis, aliquots of 2.5 × 10^5^ TK6 cells were exposed to the concentrations determined from previous assays for 26 h in the absence of S9 mix, or for 3 h in the presence of S9 mix followed by a 23 h recovery period.

Metodesnitazene and etodesnitazene were tested at 0, 12.5 and 25 μM and at 0, 12.5, 25 and 50 μM, respectively; under metabolic activation, the same concentrations were applied for metodesnitazene, while etodesnitazene was tested at 0, 25 and 50 μM.

**Figure 11:**
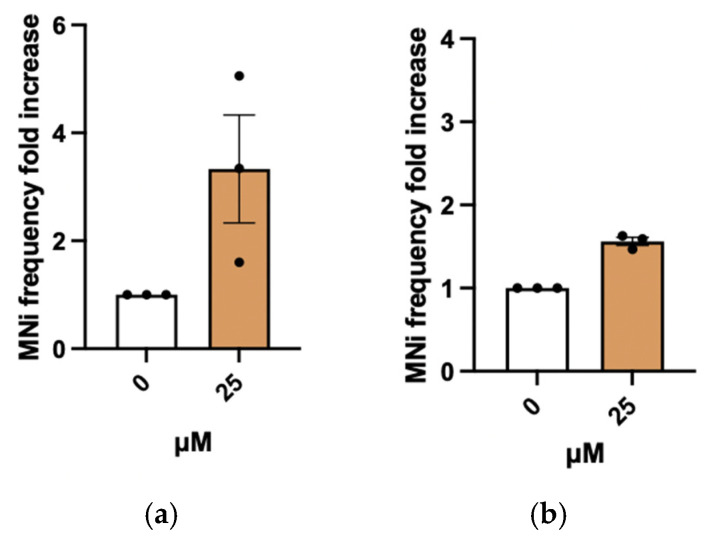
Representative scatter plot for MNi frequency fold increase on TK6 cells after (**a**) metodesnitazene and (**b**) etodesnitazene treatment at concentrations of 0 and 25 μM. The graph illustrates the distribution of MN frequency values across three independent experiments. Data were analyzed by Repeated Measures ANOVA followed by Bonferroni post-test.

Aliquots of 5 × 10^5^ cells were then processed using an automated flow cytometry protocol (FCM) developed by Lenzi et al. [31], in which cells were first lysed and treated with RNase to degrade cytoplasmic and nuclear RNA, and then SYTOX Green was added to stain nuclei and MNi. For each sample, 5000 nuclei were acquired. In flow cytometry analyses, events were first gated based on FSC/SSC parameters to exclude doublets and debris. Subsequently, nuclei and MNi were discriminated according to size and green fluorescence intensity. MNi frequency is expressed as n° of MNi/5000 nuclei. Figure 11 shows an example of two final scatter plot obtained from the analysis.

#### 4.4.6. Measurement of Intracellular ROS Levels

To assess ROS generation, H_2_O_2_ levels were measured using a CL cell-based assay described by Calabria et al. [32]. Briefly, the assay employs a CL probe that reacts selectively with intracellular H_2_O_2_, producing a luminescent signal detectable at 540 nm.

Cells were treated with metodesnitazene (25 μM) or etodesnitazene (50 μM), and measurements were performed after 3 and 6 h of exposure. At each time point, 100 μL aliquots containing 2.5 × 10^5^ cells were incubated with 100 μL of the CL probe (final concentration of 5 μM) for 20 min at 37 °C to allow probe uptake. Menadione (25 μM) was used as a positive control, while untreated cells served as negative controls.

Chemiluminescence was recorded for 40 min using a Varioskan™ LUX Multimode Microplate Reader (Thermo Fisher Scientific, Roskilde, Denmark). The entire assay was conducted at 37 °C. Signals were quantified as the integrated emission (area under the curve, AUC) over the 20–40 min interval following background subtraction, as previously described [32].

#### 4.4.7. Flow Cytometry

All analyses were performed by FCM using a Guava easyCyte 5HT Flow Cytomer equipped with a class IIIb laser operating at a wavelength of 488 nm (Cytek Bioscences Inc. Fremont, CA, USA).

#### 4.4.8. Statistical Analysis

Each experiment was conducted in triplicate, and the results are presented as mean ± Standard Error of the Mean (SEM) of three independent experiments. Data normalization was performed individually within each independent experimental run to inherently control for inter-experimental variations (batch effects). Specifically, for cytotoxicity and cytostasis assays, raw values were normalized against the concurrent internal negative control, conventionally set to 100%, and expressed as percentages. For genotoxic and apoptosis evaluations, data were normalized to the concurrent internal negative control, set to 1, and expressed as fold increase. Statistical significance was analyzed by Repeated Measures one-way analysis of variance (ANOVA), followed by Dunnett’s or Bonferroni’s post hoc test carried out in Prism 9.0 (GraphPad Software, 10.3.1, Boston, MA, USA). The results were considered statistically significant for *p* < 0.05.

## Data Availability

The original contributions presented in the study are included in the article. Further inquiries can be directed to the corresponding author.

## Abbreviations

The following abbreviations are used in this manuscript:

NPS	New Psychoactive Substances
TK6	Human lymphoblastoid cells
MNi	Micronuclei
FCM	Flow Cytometry
ROS	Reactive Oxygen Species
UNODC	United Nations Office on Drugs and Crime
NSO	New Synthetic Opioids
MOR	Mu-Opioid Receptor
DOR	Delta-Opioid Receptor
KOR	Kappa-Opioid Receptor
CIBA	Chemische Industrie Basel Aktiengesellschaft
DEA	Drug Enforcement Administration
OECD	Organisation for Economic Co-operation and Development
DNA	Deoxyribonucleic Acid
RPD	Relative Population Doubling
SEM	Standard Error of the Mean
ANOVA	Analysis of Variance
PI	Propidium Iodide
S9	Supernatant 9—Exogenous metabolizing systems
CL	Chemiluminescent
AUC	Area Under the Curve
SD	Standard Deviation
EDTA	Ethylenediaminetetraacetic acid
PS	Penicillin-Streptomycin solution
RPMI	Roswell Park Memorial Institute
FBS	Fetal Bovine Serum
PD	Population Doubling
RNA	Ribonucleic Acid

## References

[1] Marandiuc I.-M., Docea A.O., Hîrjău A.C., Avram O.R., Matei C.-S., Tsatsakis A., Arsene A.-L. (2025). New Psychoactive Substances: A Multidisciplinary Review of Challenges and Their Diverse Character. DARU J. Pharm. Sci..

[2] Gonçalves J., Luís Â., Gallardo E., Duarte A.P. (2021). Psychoactive Substances of Natural Origin: Toxicological Aspects, Therapeutic Properties and Analysis in Biological Samples. Molecules.

[3] Data Visualisations—Public. https://www.unodc.org/LSS/Page/NPS/DataVisualisations.

[4] Taflaj B., La Maida N., Tittarelli R., Di Trana A., D’Acquarica I. (2024). New Psychoactive Substances Toxicity: A Systematic Review of Acute and Chronic Psychiatric Effects. Int. J. Mol. Sci..

[5] Klingberg J., Keen B., Cawley A., Pasin D., Fu S. (2022). Developments in High-Resolution Mass Spectrometric Analyses of New Psychoactive Substances. Arch. Toxicol..

[6] Zawilska J.B., Adamowicz P., Kurpeta M., Wojcieszak J. (2023). Non-Fentanyl New Synthetic Opioids—An Update. Forensic Sci. Int..

[7] Kadem S.N., Arslan Z., Turkmen Z. (2025). A New Synthetic Opioid Threat: A Comprehensive Review on MT-45. Forensic Sci. Int..

[8] Lovrecic B., Lovrecic M., Gabrovec B., Carli M., Pacini M., Maremmani A.G.I., Maremmani I. (2019). Non-Medical Use of Novel Synthetic Opioids: A New Challenge to Public Health. Int. J. Environ. Res. Public Health.

[9] Giorgetti A., Pascali J., Fais P., Pelletti G., Gabbin A., Franchetti G., Cecchetto G., Viel G. (2021). Molecular Mechanisms of Action of Novel Psychoactive Substances (NPS). A New Threat for Young Drug Users with Forensic-Toxicological Implications. Life.

[10] Albores-Garcia D., Cruz S.L. (2023). Fentanyl and Other New Psychoactive Synthetic Opioids. Challenges to Prevention and Treatment. Rev. Investig. Clín..

[11] Ujváry I., Christie R., Evans-Brown M., Gallegos A., Jorge R., De Morais J., Sedefov R. (2021). DARK Classics in Chemical Neuroscience: Etonitazene and Related Benzimidazoles. ACS Chem. Neurosci..

[12] News: February 2026—Increasing Nitazene and Orphine Analogues (Synthetic Opioids) and the Implications for the Use of Test Strips. https://www.unodc.org/LSS/Announcement/Details/e69b2ff5-5b91-4eea-8e1f-802ca7ad5080.

[13] Pergolizzi J., Raffa R., LeQuang J.A.K., Breve F., Varrassi G. (2023). Old Drugs and New Challenges: A Narrative Review of Nitazenes. Cureus.

[14] EU Drug Market: NPS (EUDA). https://www.euda.europa.eu/publications/eu-drug-markets/new-psychoactive-substances/introduction_en.

[15] Pereira J.R.P., Quintas A., Neng N.R. (2025). Nitazenes: The Emergence of a Potent Synthetic Opioid Threat. Molecules.

[16] Vandeputte M.M., Stove C.P. (2025). Navigating Nitazenes: A Pharmacological and Toxicological Overview of New Synthetic Opioids with a 2-Benzylbenzimidazole Core. Neuropharmacology.

[17] Roberts A., Korona-Bailey J., Mukhopadhyay S. (2022). Notes from the Field: Nitazene-Related Deaths—Tennessee, 2019–2021. Morb. Mortal. Wkly. Rep..

[18] De Vrieze L.M., Walton S.E., Pottie E., Papsun D., Logan B.K., Krotulski A.J., Stove C.P., Vandeputte M.M. (2024). In Vitro Structure–Activity Relationships and Forensic Case Series of Emerging 2-Benzylbenzimidazole ‘Nitazene’ Opioids. Arch. Toxicol..

[19] Caprari C., Ferri E., Rossetti P., Gregori A., Citti C., Cannazza G. (2025). The Emergence of Nitazenes: A New Chapter in the Synthetic Opioid Crisis. Arch. Toxicol..

[20] Vandeputte M.M., Glatfelter G.C., Walther D., Layle N.K., St. Germaine D.M., Ujváry I., Iula D.M., Baumann M.H., Stove C.P. (2024). Characterization of Novel Nitazene Recreational Drugs: Insights into Their Risk Potential from in Vitro µ-Opioid Receptor Assays and in Vivo Behavioral Studies in Mice. Pharmacol. Res..

[21] Kozell L.B., Eshleman A.J., Wolfrum K.M., Swanson T.L., Bloom S.H., Benware S., Schmachtenberg J.L., Schutzer K.A., Schutzer W.E., Janowsky A. (2024). Pharmacologic Characterization of Substituted Nitazenes at μ, κ, and Δ Opioid Receptors Suggests High Potential for Toxicity. J. Pharmacol. Exp. Ther..

[22] Vandeputte M.M., Van Uytfanghe K., Layle N.K., St. Germaine D.M., Iula D.M., Stove C.P. (2021). Synthesis, Chemical Characterization, and μ-Opioid Receptor Activity Assessment of the Emerging Group of “Nitazene” 2-Benzylbenzimidazole Synthetic Opioids. ACS Chem. Neurosci..

[23] World Health Organization (2023). WHO Expert Committee on Drug Dependence: Forty-Fifth Report.

[24] Kurach Ł., Chłopaś-Konowałek A., Budzyńska B., Zawadzki M., Szpot P., Boguszewska-Czubara A. (2021). Etazene Induces Developmental Toxicity in Vivo Danio Rerio and in Silico Studies of New Synthetic Opioid Derivative. Sci. Rep..

[25] Taoussi O., Berardinelli D., Zaami S., Tavoletta F., Basile G., Kronstrand R., Auwärter V., Busardò F.P., Carlier J. (2024). Human Metabolism of Four Synthetic Benzimidazole Opioids: Isotonitazene, Metonitazene, Etodesnitazene, and Metodesnitazene. Arch. Toxicol..

[26] Vitrano A., Di Giorgi A., Abbate V., Basile G., La Maida N., Pichini S., Di Trana A. (2024). Evaluation of Short-Term Stability of Different Nitazenes Psychoactive Opioids in Dried Blood Spots by Liquid Chromatography-High-Resolution Mass Spectrometry. Int. J. Mol. Sci..

[27] Federal Register/Vol. 89, No. 207/Friday, October 25, 2024/Rules and Regulations: (722922011-001) 2011. https://www.govinfo.gov/content/pkg/FR-2024-10-25/pdf/2024-23881.pdf.

[28] Federal Register/Vol. 89, No. 71/Thursday, April 11, 2024/Rules and Regulations. https://www.govinfo.gov/content/pkg/FR-2024-04-11/pdf/2024-07514.pdf.

[29] Rombolà F., Bartoletti S., Bilel S., Hrelia P., Marti M., Lenzi M. (2025). In Vitro Cytotoxic and Genotoxic Evaluation of Nitazenes, a Potent Class of New Synthetic Opioids. J. Xenobiotics.

[30] OECD (2023). Test No. 487: In Vitro Mammalian Cell Micronucleus Test. OECD Guidelines for the Testing of Chemicals.

[31] Lenzi M., Cocchi V., Hrelia P. (2018). Flow Cytometry vs. Optical Microscopy in the Evaluation of the Genotoxic Potential of Xenobiotic Compounds. Cytom. Part B Clin..

[32] Calabria D., Guardigli M., Mirasoli M., Punzo A., Porru E., Zangheri M., Simoni P., Pagnotta E., Ugolini L., Lazzeri L. (2020). Selective Chemiluminescent TURN-ON Quantitative Bioassay and Imaging of Intracellular Hydrogen Peroxide in Human Living Cells. Anal. Biochem..

[33] Srivastava R., Mishra N., Singh U.M., Srivastava R. (2016). Genotoxicity: Mechanisms and Its Impact on Human Diseases. Octa J. Biosci..

[34] Gasperini S., Bilel S., Cocchi V., Marti M., Lenzi M., Hrelia P. (2022). The Genotoxicity of Acrylfentanyl, Ocfentanyl and Furanylfentanyl Raises the Concern of Long-Term Consequences. Int. J. Mol. Sci..

[35] Lenzi M., Gasperini S., Bilel S., Corli G., Rombolà F., Hrelia P., Marti M. (2025). New Synthetic Opioids: What Do We Know About the Mutagenicity of Brorphine and Its Analogues?. Int. J. Mol. Sci..

[36] Luukkonen J., Liimatainen A., Höytö A., Juutilainen J., Naarala J. (2011). Pre-Exposure to 50 Hz Magnetic Fields Modifies Menadione-Induced Genotoxic Effects in Human SH-SY5Y Neuroblastoma Cells. PLoS ONE.

[37] Martins E.A.L., Meneghini R. (1990). DNA Damage and Lethal Effects of Hydrogen Peroxide and Menadione in Chinese Hamster Cells: Distinct Mechanisms Are Involved. Free Radic. Biol. Med..

[38] Woods J.A., Young A.J., Gilmore I.T., Morris A., Bilton R.F. (1997). Measurement of Menadione-Mediated DNA Damage in Human Lymphocytes Using the Comet Assay. Free Radic. Res..

[39] Cojocel C., Novotny L., Vachalkova A. (2006). Mutagenic and Carcinogenic Potential of Menadione. Neoplasma.

[40] Seager A.L., Shah U.-K., Mikhail J.M., Nelson B.C., Marquis B.J., Doak S.H., Johnson G.E., Griffiths S.M., Carmichael P.L., Scott S.J. (2012). Pro-Oxidant Induced DNA Damage in Human Lymphoblastoid Cells: Homeostatic Mechanisms of Genotoxic Tolerance. Toxicol. Sci..

[41] Vijayaraghavalu D.S. (2010). Genotoxicity: From Basics to Regulatory Insights. Mutat. Res. Rev. Mutat. Res..

[42] Li X., Chen S., Guo X., Wu Q., Seo J.-E., Guo L., Manjanatha M.G., Zhou T., Witt K.L., Mei N. (2020). Development and Application of TK6-Derived Cells Expressing Human Cytochrome P450s for Genotoxicity Testing. Toxicol. Sci..

